# Transcription profile data of phorbol esters biosynthetic genes during developmental stages in *Jatropha curcas*

**DOI:** 10.1016/j.dib.2018.03.061

**Published:** 2018-03-21

**Authors:** Nurul Jadid, Rizal Kharisma Mardika, Kristanti Indah Purwani, Erlyta Vivi Permatasari, Indah Prasetyowati, Mohammad Isa Irawan

**Affiliations:** a Department of Biology, Institut Teknologi Sepuluh Nopember, Surabaya 60111, Indonesia; b Department of Mathematics, Institut Teknologi Sepuluh Nopember, Surabaya 60111, Indonesia

**Keywords:** Casbene synthase, Diterpenoid, Geranylgeranyl diphosphate synthase, *Jatropha curcas*, Phorbol esters

## Abstract

*Jatropha curcas* is currently known as an alternative source for biodiesel production. Beside its high free fatty acid content, *J. curcas* also contains typical diterpenoid-toxic compounds of Euphorbiaceae plant namely phorbol esters. This article present the transcription profile data of genes involved in the biosynthesis of phorbol esters at different developmental stages of leaves, fruit, and seed in *Jatropha curcas*. Transcriptional profiles were analyzed using reverse transcription-polymerase chain reaction (RT-PCR). We used two genes including *GGPPS* (Geranylgeranyl diphospate synthase), which is responsible for the formation of common diterpenoid precursor (GGPP) and *CS* (Casbene Synthase), which functions in the synthesis of casbene. Meanwhile, *J. curcas Actin* (*ACT*) was used as internal standard. We demonstrated dynamic of *GGPPS* and *CS* expression among different stage of development of leaves, fruit and seed in *Jatropha*.

**Specifications Table**

**Table:** 

Subject area	*Biology*
More specific subject area	*Molecular plant biology, plant physiology*
Type of data	*Figures and text*
How data was acquired	*cDNA synthesis, RT-PCR and image analysis*
Data format	*Analyzed*
Experimental factors	*Genes involved in the biosynthesis of phorbol esters were GGPPS and CS. The expression of each gene was analyzed using reverese-transcriptase PCR (RT-PCR).*
Experimental features	*Samples consisted of three plant organs including leaves, endosperm and fruit (pericarp) in two different developmental stages (young and mature stages). All samples were subjected to total RNA extraction followed by cDNA synthesis. The cDNA obtained was then amplified using specific GGPPS and CS primers. ACT (actin) was used as internal standard.*
Data source location	*Department of Biology, Institut Teknologi Sepuluh Nopember, Surabaya, Indonesia*
Data accessibility	*The data are available with this article*

**Value of the data**

• The transcription profile of *JcGGPPS* and *JcCS* data of the *J. curcas* demonstrate the dynamic expression of the genes in different plant organs at distinct developmental stages.
• The data are useful to be combined with biochemical analysis to determine in which part of plant organ the phorbol esters are accumulated.
• Determination of the expression of both genes might contribute for further study to understand the relationship between phorbol esters biosynthesis and plant development.

## 1 Data

**Fig. 1:**
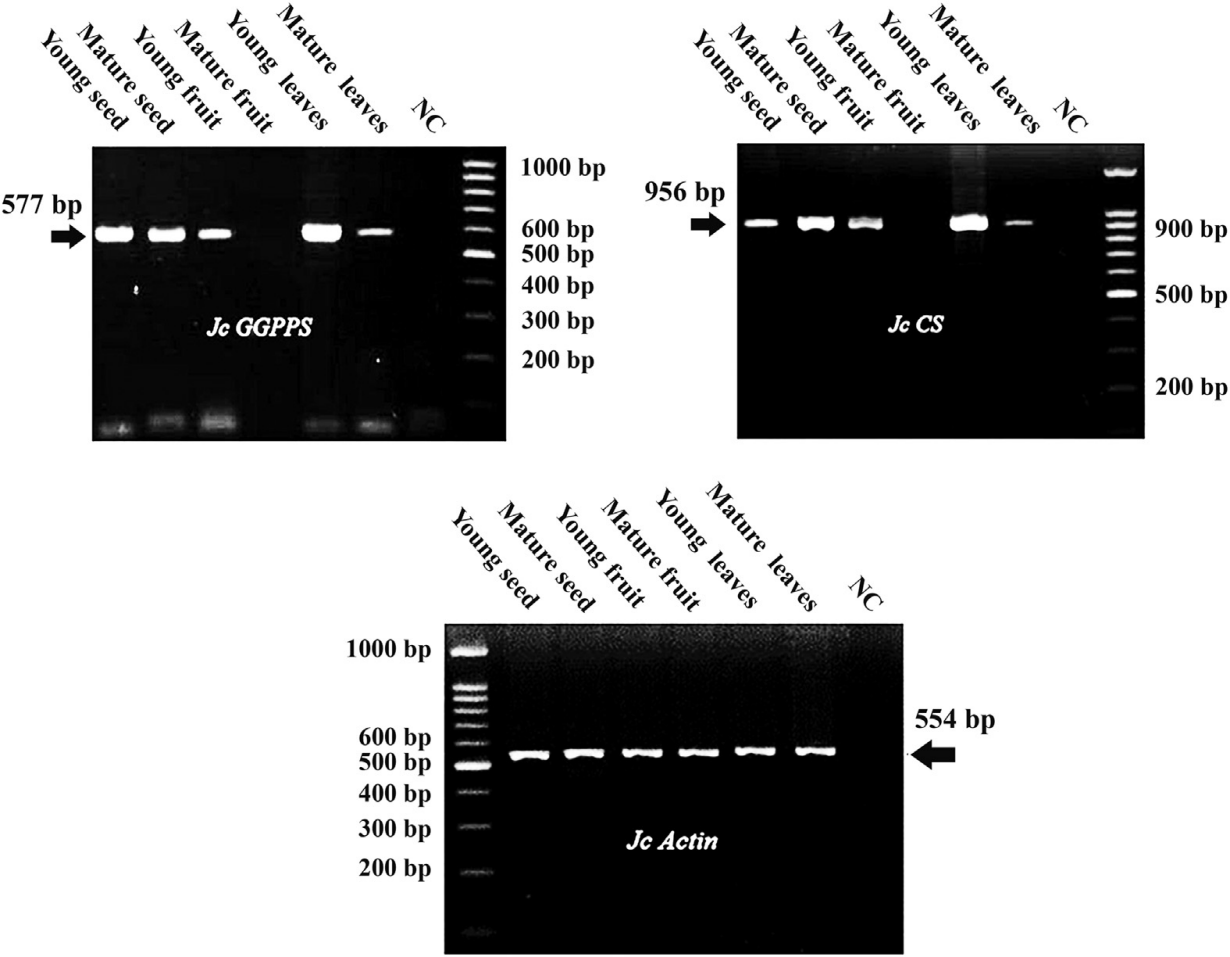
Amplification of *JcGGPPS, JcCS* and *JcActin* using specific primers. Amplicons obtained for *JcGGPPS, JcCS* and *JcActin* were 577 bp, 956 bp and 554 bp, respectively.

**Fig. 2:**
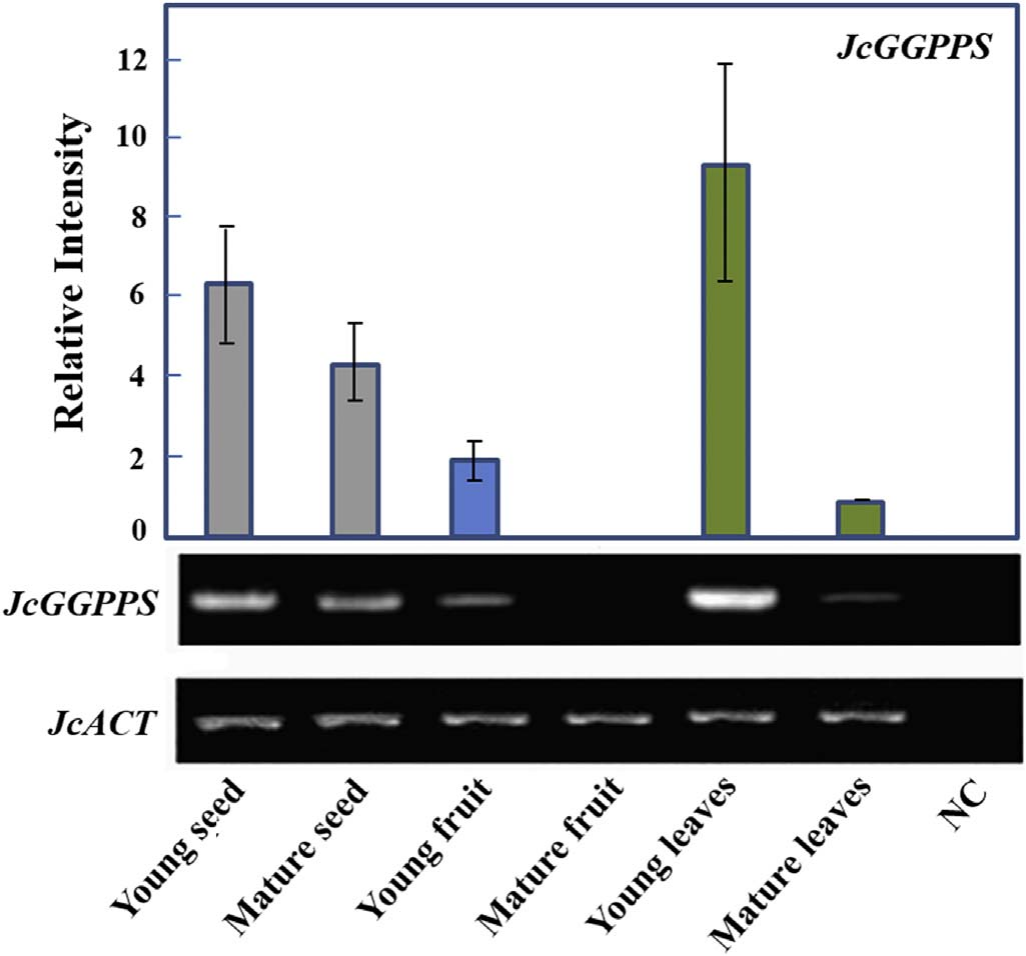
Transcription profile of *JcGGPPS* gene in different organs and developmental stages of *Jatropha curcas.* The expression profiles are demonstrated as ratio relative to matured leaves using ImageJ software. The gel electrophoresis figure is representative of triplicate. Grey, blue and green bars represent relative intensity of *JcGGPPS* from seed, fruit and leaves respectively. NC; negative control. (For interpretation of the references to color in this figure legend, the reader is referred to the web version of this article.)

**Fig. 3:**
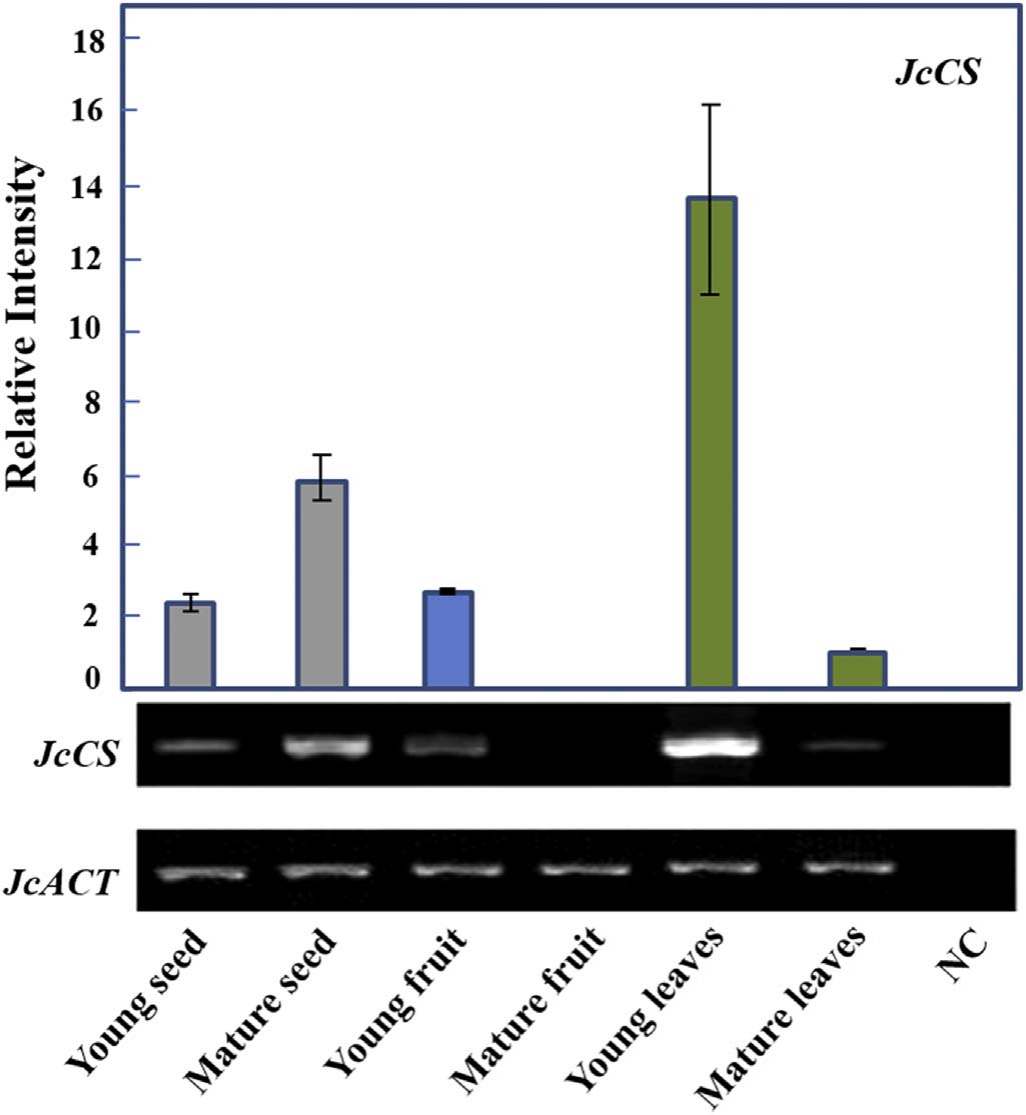
Expression of *JcCS* gene in different organs and developmental stages of *Jatropha curcas.* The expression profiles are demonstrated as ratio relative to matured leaves using ImageJ software. The gel electrophoresis figure is representative of triplicate. Grey, blue and green bars represent relative intensity of *JcCS* from seed, fruit and leaves respectively. NC; negative control. (For interpretation of the references to color in this figure legend, the reader is referred to the web version of this article.)

*Jatropha curcas* - a species of Euphorbiaceae family - is generally found in the tropical asian countries [1]. Jatropha is currently cultivated and is importantly used for alternative biodiesel development throughout those regions. Like other Euphorbiaceae plants, *J. curcas* is characterized by the presence of toxic compounds, including phorbol esters [2]. Here, we demonstrate the transcription profile of *GGPPS* and *CS* as key genes involved in the biosynthesis of phorbol esters [3]. The profile was analyzed semi quantitatively using Reverese Transcription-Polymerase Chain Reaction (RT-PCR) at different stages of leaves, seeds (endosperm) and fruit (exocarp) development (young and matured organs). Young fruit and seed were collected at 29 days after pollination (dap). Meanwhile, the matured exocarp and seed were at 35 and 41 dap, respectively. Fig. 1 shows different DNA fragments with varying sizes representing *JcGGPPS, JcCS* and *JcACT* (577 bp, 956 bp and 554 bp, respectively). Fig. 2, Fig. 3 demonstrate the transcription profile of *JcGGPPS* dan *JcCS* genes in different organs and developmental stages of *J. curcas*.

## 2 Experimental design, materials, and methods

### 2.1 Collection of plant materials

Fruits (exocarp), seeds (endosperm) and leaves of *J. curcas* were obtained from Purwodadi-Botanical Garden, The Indonesian Institute of Science (LIPI), Indonesia. No specific regulations were required since the samples did not involve endangered/ protected species. Vegetative samples (leaves) were taken from young and matured tissues. Whereas, generative samples including fruit and seed were collected at different developmental stages. Young fruit and seed were taken from sample at 29 days after pollination (dap). Meanwhile, matured fruit were characterized by yellowish color of the exocarp and collected at 35 dap. Matured seed were taken from fruit at 41 dap. All samples (100 mg) were then powdered using liquid nitrogen for being used in total ribonucleic acid (RNA) extraction.

### 2.2 Total RNA extraction and first strand cDNA synthesis

All fine powder samples were subjected to total RNA extraction using Plant total RNA mini kit (Geneaid) according to the manufacturer's instructions. The concentration and also the quality of extracted RNA were measured using NanoDrop^™^ 2000 UV–vis Spectrophotometer and directly stored at −20 °C before being used for complementary deoxyribonucleic acid (cDNA) synthesis. 1 µg total RNA from the six tested samples (fruits, seeds amd leaves at different developmental stages) were used for cDNA synthesis using AffinityScript cDNA synthesis (Agilent Genomics) according to the manufacturer's protocols.

### 2.3 Amplification of *JcGGPPS* and *JcCS*

The cDNA obtained previously were then subjected to a conventional amplification (end-point PCR) as previously described [4] using KAPA2G Fast ReadyMix PCR Kit (Kapa Biosystems) in a final reaction volume of 25 µL, which consist of 3 µL cDNA as a template, 0.5 µM of each gene-specific primers and 1×KAPA2G Fast ReadyMix. The genes used in this study were *JcGGPPS* (GenBank accession number: GU585938) and *JcCS* (GenBank accession number: AB687998). A Jatropha actin gene (*JcACT*, GenBank accession number: JQ806331) was used as internal standard. All gene-specific primers were previously designed using open access primer designing tools from the National Center for Biotechnology Information (NCBI) (“http://www.ncbi.nlm.nih.gov/tools/primer-blast/”). The primers used for amplification are 5’-GTCCTGAACTCCCATTTAACCAC-3’ and 5’-CTCAGTTATGACTCGAACCACTC-3’ for *JcGGPPS*; 5’-CTACTGTATGGGGCGATCGACTTGC-3’ and 5’- CATGTAATTTGGCAGTTGGTCGAC-3’ for *JcCS* and 5’- GGATATTCAGCCCCTGGTTT-3’ and 5’- CATCAGTGAGATCACGACCA-3’ for *JcACT.* The amplification conditions were as follows: 3 min at 95 °C; 40 cycles of 15 s at 95 °C, 15 s at 60 °C and 15 s at 72 °C; followed by 15 s at 72 °C. Negative controls were included in all experiments. PCR amplicons were detected and visualized using 2% gel electrophoresis containing 1×Tris-Borate-EDTA (TBE) buffer and 10 mg/mL ethidium bromide (Fig. 1). All data obtained in this study was analyzed descriptively where electrophoresis results and band intensities were recorded and measured using ImageJ (National Institute of Health) analysis according to the method described previously [4]. The expression levels were presented as relative intensity to matured leaves, which was set to 1 (Fig. 2, Fig. 3).

## References

[1] Nakano Y., Ohtani M., Polsri W., Usami T., Sambongi K., Demura T. (2012). Characterization of the casbene synthase homolog from Jatropha (Jatropha curcas L.). Plant Biotechnol..

[2] Makkar H.P.S., Becker K., Sporer F., Wink M. (1997). Studies on nutritive potential and toxic constituents of different provenances of Jatropha curcas. J. Agric. Food Chem..

[3] N. Jadid, R.K. Mardika, T. Nurhidayati, M.I. Irawan, Reverse transcription-PCR analysis of geranylgeranyl diphosphate synthase (*JcGGPPS*) in *Jatropha curcas L. and in silico analysis of casbene synthase (JcCS) among Euphorbiaceae*, AIP Conference Proceedings, 17440, 2, 0042, 10.1063/1.4953516, 2016.

[4] Zhang B., Chen K., Bowen J., Allan A., Espley R., Karunairetnam S., Ferguson I. (2006). Differential expression within the LOX gene family in ripening kiwifruit. J. Exp. Bot..

